# Supplementary material for: Multi‐dataset identification of innovative feature genes and molecular mechanisms in keratoconus
Source: J Cell Mol Med. 2024 Sep 19;28(18):e70079. doi: 10.1111/jcmm.70079 (PMC11412914; doi:10.1111/jcmm.70079)
Supplement: Supplementary file 2 — Material S2. [file JCMM-28-e70079-s002.zip › 7.GSEA analysis/2.ATOH7/KEGG_EPITHELIAL_CELL_SIGNALING_IN_HELICOBACTER_PYLORI_INFECTION.html]

Details for gene set KEGG\_EPITHELIAL\_CELL\_SIGNALING\_IN\_HELICOBACTER\_PYLORI\_INFECTION[GSEA]

|  || Dataset | ATOH7.group.cls#h\_versus\_l |
| Phenotype | group.cls#h\_versus\_l |
| Upregulated in class | h |
| GeneSet | KEGG\_EPITHELIAL\_CELL\_SIGNALING\_IN\_HELICOBACTER\_PYLORI\_INFECTION |
| Enrichment Score (ES) | 0.48980755 |
| Normalized Enrichment Score (NES) | 1.6610496 |
| Nominal p-value | 0.02661597 |
| FDR q-value | 0.226774 |
| FWER p-Value | 0.473 |
Table: GSEA Results Summary

  

Fig 1: Enrichment plot: KEGG\_EPITHELIAL\_CELL\_SIGNALING\_IN\_HELICOBACTER\_PYLORI\_INFECTION      
 Profile of the Running ES Score & Positions of GeneSet Members on the Rank Ordered List

  

| SYMBOL | TITLE | RANK IN GENE LIST | RANK METRIC SCORE | RUNNING ES | CORE ENRICHMENT || 1 | JAM2 | na | 98 | 0.775 | 0.0991 | Yes |
| 2 | CXCL1 | na | 687 | 0.500 | 0.1479 | Yes |
| 3 | JAM3 | na | 846 | 0.472 | 0.2053 | Yes |
| 4 | CXCR1 | na | 1036 | 0.442 | 0.2580 | Yes |
| 5 | PTPRZ1 | na | 1319 | 0.406 | 0.3032 | Yes |
| 6 | ATP6V1G2 | na | 1480 | 0.388 | 0.3497 | Yes |
| 7 | IGSF5 | na | 1544 | 0.382 | 0.3981 | Yes |
| 8 | SRC | na | 4033 | 0.235 | 0.3572 | Yes |
| 9 | CXCL8 | na | 4857 | 0.206 | 0.3605 | Yes |
| 10 | PAK1 | na | 4910 | 0.204 | 0.3858 | Yes |
| 11 | MAP3K14 | na | 4980 | 0.202 | 0.4104 | Yes |
| 12 | ATP6V1E2 | na | 5651 | 0.181 | 0.4149 | Yes |
| 13 | MET | na | 6191 | 0.165 | 0.4211 | Yes |
| 14 | RELA | na | 6248 | 0.164 | 0.4410 | Yes |
| 15 | LYN | na | 6447 | 0.158 | 0.4560 | Yes |
| 16 | MAPK12 | na | 6613 | 0.154 | 0.4715 | Yes |
| 17 | ATP6V1B1 | na | 6672 | 0.152 | 0.4898 | Yes |
| 18 | CCL5 | na | 7689 | 0.126 | 0.4770 | No |
| 19 | NFKB1 | na | 8680 | 0.105 | 0.4622 | No |
| 20 | NFKBIA | na | 10503 | 0.073 | 0.4193 | No |
| 21 | ATP6V0E2 | na | 10518 | 0.073 | 0.4286 | No |
| 22 | ADAM17 | na | 10662 | 0.071 | 0.4338 | No |
| 23 | ATP6V1C2 | na | 10800 | 0.069 | 0.4389 | No |
| 24 | ADAM10 | na | 11689 | 0.057 | 0.4207 | No |
| 25 | ATP6V1H | na | 11715 | 0.056 | 0.4274 | No |
| 26 | ATP6V1E1 | na | 12279 | 0.049 | 0.4176 | No |
| 27 | EGFR | na | 12485 | 0.046 | 0.4178 | No |
| 28 | ATP6V1A | na | 13508 | 0.035 | 0.3930 | No |
| 29 | ATP6V1G1 | na | 13709 | 0.033 | 0.3916 | No |
| 30 | ATP6V0C | na | 14348 | 0.027 | 0.3767 | No |
| 31 | ATP6V1F | na | 14695 | 0.024 | 0.3699 | No |
| 32 | NOD1 | na | 15478 | 0.017 | 0.3495 | No |
| 33 | IKBKG | na | 15619 | 0.016 | 0.3476 | No |
| 34 | ATP6V1G3 | na | 15937 | 0.013 | 0.3402 | No |
| 35 | RAC1 | na | 16331 | 0.010 | 0.3302 | No |
| 36 | ATP6V0A1 | na | 16474 | 0.009 | 0.3272 | No |
| 37 | GIT1 | na | 16476 | 0.009 | 0.3284 | No |
| 38 | JUN | na | 16587 | 0.008 | 0.3263 | No |
| 39 | ATP6V0E1 | na | 16834 | 0.006 | 0.3200 | No |
| 40 | ATP6V1C1 | na | 16908 | 0.005 | 0.3185 | No |
| 41 | ATP6V1B2 | na | 17084 | 0.004 | 0.3140 | No |
| 42 | MAPK8 | na | 17140 | 0.003 | 0.3128 | No |
| 43 | F11R | na | 17825 | -0.003 | 0.2934 | No |
| 44 | CDC42 | na | 17874 | -0.003 | 0.2924 | No |
| 45 | ATP6AP1 | na | 18125 | -0.005 | 0.2859 | No |
| 46 | ATP6V0B | na | 18789 | -0.011 | 0.2682 | No |
| 47 | ATP6V1D | na | 18877 | -0.012 | 0.2672 | No |
| 48 | ATP6V0D1 | na | 20156 | -0.023 | 0.2334 | No |
| 49 | CHUK | na | 20245 | -0.024 | 0.2341 | No |
| 50 | CSK | na | 20669 | -0.028 | 0.2256 | No |
| 51 | MAPK10 | na | 20989 | -0.032 | 0.2205 | No |
| 52 | MAP2K4 | na | 21265 | -0.035 | 0.2171 | No |
| 53 | PTPN11 | na | 22613 | -0.050 | 0.1848 | No |
| 54 | MAPK14 | na | 23324 | -0.059 | 0.1721 | No |
| 55 | IKBKB | na | 23356 | -0.060 | 0.1791 | No |
| 56 | HBEGF | na | 23932 | -0.069 | 0.1715 | No |
| 57 | MAPK13 | na | 23983 | -0.069 | 0.1792 | No |
| 58 | PLCG2 | na | 24577 | -0.078 | 0.1724 | No |
| 59 | TJP1 | na | 24758 | -0.081 | 0.1779 | No |
| 60 | MAPK9 | na | 24826 | -0.082 | 0.1868 | No |
| 61 | CASP3 | na | 24828 | -0.083 | 0.1976 | No |
| 62 | PLCG1 | na | 25238 | -0.090 | 0.1976 | No |
| 63 | MAPK11 | na | 25479 | -0.094 | 0.2030 | No |
| 64 | ATP6V0A4 | na | 26338 | -0.111 | 0.1929 | No |
| 65 | ATP6V0D2 | na | 27974 | -0.150 | 0.1655 | No |
| 66 | CXCR2 | na | 30340 | -0.223 | 0.1266 | No |
Table: GSEA details [plain text format]

  

Fig 2: KEGG\_EPITHELIAL\_CELL\_SIGNALING\_IN\_HELICOBACTER\_PYLORI\_INFECTION      
 Blue-Pink O' Gram in the Space of the Analyzed GeneSet

  

Fig 3: KEGG\_EPITHELIAL\_CELL\_SIGNALING\_IN\_HELICOBACTER\_PYLORI\_INFECTION: Random ES distribution      
 Gene set null distribution of ES for **KEGG\_EPITHELIAL\_CELL\_SIGNALING\_IN\_HELICOBACTER\_PYLORI\_INFECTION**
